# Extracellular vesicles released from Marek’s disease virus-transformed T-cells impact immune cell proliferation

**DOI:** 10.1099/jgv.0.002191

**Published:** 2025-12-04

**Authors:** Laëtitia Trapp-Fragnet, Julien Burgaud-Gaillard, Valérie Labas, Sylvie Rémy, Ana-Paula Texeira-Gomes, Caroline Denesvre

**Affiliations:** 1 Equipe Biologie des Virus Aviaires, UMR1282 ISP, INRAE, Nouzilly, France; 2 Plate-Forme IBiSA des Microscopies, PPF ASB, Université de Tours and CHRU de Tours, Tours, France; 3 INRAE, CNRS, Université de Tours, PRC, Nouzilly, France; 4 PIXANIM, Université de Tours, CHU de Tours, INRAE, Nouzilly, France

**Keywords:** chicken lymphocytes, exosome, extracellular vesicles, lymphomagenesis, Marek’s disease virus, proliferation

## Abstract

Marek’s disease virus (MDV) is an alphaherpesvirus responsible for the development of T-cell lymphoma in chickens. Despite the identification of several pro-oncogenic viral molecules encoded by MDV, the processes leading to tumourigenesis remain poorly understood. Extracellular vesicles (EVs) are important mediators of intercellular communication, carrying bioactive molecules that can elicit profound physiological changes in recipient cells. Tumour cells can release significant amounts of EVs, which influence tumour development and growth, metastatic processes and resistance to cancer therapies. These EVs favour cancer cells to evade the immune response, particularly by establishing an immunosuppressive microenvironment. Here, we investigated whether EVs produced by MDV-transformed T lymphocytes affect the proliferation of avian immune cells, a determining feature in neoplastic processes. EVs were purified from an MDV-transformed cell line cultured *in vitro*. Using a proteomic approach, we confirmed the presence of specific markers and identified a panel of cellular proteins enriched in these EVs. Notably, no viral proteins were detected in the purified EVs. We also demonstrated that EVs are rapidly internalized by recipient chicken cells. Moreover, these EVs can induce a decrease in primary chicken B-cell proliferation, while promoting primary chicken T-cell proliferation. Our findings suggest that EVs released by MDV-transformed cells may contribute to immunosuppression and potentially facilitate lymphoma progression by enhancing T-cell proliferation.

## Introduction

Marek’s disease virus (MDV), taxonomically referred to as *Mardivirus gallidalpha2*, is a highly oncogenic alphaherpesvirus that infects chickens and causes massive economic losses to the poultry industry worldwide [1,2]. MDV elicits the rapid onset of malignant T-cell lymphomas [3] and is considered to be the most prevalent clinically diagnosed cancer in the animal kingdom. Chickens become infected through the inhalation of MDV-contaminated dander present in the environment. In the respiratory tract, B-cells are among the first cells to become infected and to support productive replication of MDV. The virus is then rapidly transported to lymphoid organs and transmitted from B-cells to activated T-cells, the ultimate target cells in which the virus replicates and subsequently establishes a latent infection. In CD4^+^ T-cells, oncogenic MDV strains induce cellular transformation, which ultimately leads to the formation of solid lymphomas, especially in the visceral organs [4,5]. Concurrently, MDV can reactivate from latently infected T-cells, which constitute a continuous source of virus production and contribute to its spread at the site of excretion, the feather follicle. Several viral genes have been identified as key players in MDV-induced lymphomagenesis, including the oncoprotein Meq, the viral telomerase RNA (vTR) subunit and other non-coding RNAs (micro-RNAs (miRNAs) and long non-coding RNAs) [6,7]. However, the mechanisms underlying MDV-induced lymphomagenesis are clearly not limited to the mere expression of these viral virulence factors and likely involve major cellular deregulations that are still poorly understood.

Next to tumour formation, MDV infection is also associated with immunosuppression (IS), which occurs in three phases: an early phase, marked by atrophy of lymphoid organs a few days after MDV infection; a later phase, associated with viral reactivation from latently infected T-cells; and a final phase, linked to lymphoproliferation [8,9]. While late-IS is still poorly understood, the early-IS induced by MDV has been well studied. We recently demonstrated that early-IS is associated with increased apoptosis in the thymus and the bursa of infected birds, along with sustained inhibition of cell proliferation specifically in the bursa [10]. Strikingly, only a small number of cells are infected in these organs, suggesting that infected cells may influence neighbouring uninfected cells through paracrine signalling.

Extracellular vesicles (EVs) released by cells emerged as a new mode of intercellular communication. EVs include three types of vesicles that differ in size, biogenesis and composition: (i) apoptotic bodies (800 to 5,000 nm) derived from cells undergoing apoptosis; (ii) microvesicles (MVs) (50 to 1,000 nm) generated by direct budding from the plasma membrane; and (iii) exosomes (EXOs) (30 to 100 nm) formed by invagination of the membrane composing multivesicular bodies (MVB) and released into the extracellular space upon fusion of the MVB with the plasma membrane [11]. EVs mediate the exchange of proteins and genetic material (e.g. DNA, mRNA and miRNAs) derived from parental cells, which can alter the physiological state of the recipient cells. EVs are produced by most eukaryotic cells, and their release is often exacerbated under pathological conditions, particularly during cancer development. Extracellular vesicles released from cancer cells can profoundly affect the tumour microenvironment by influencing neighbouring cells (stromal cells, immune cells etc.), which in turn promotes important tumourigenic events, including tumour growth, invasion, metastasis and resistance to cancer therapies [12,13]. Tumour-derived exosomes (TEX) can promote the transformation of normal cells by transferring growth factors and oncogenes [e.g. Epithelial Growth Factor (EGFR) and Transforming Growth Factor (TGF-*β)*], as well as non-coding RNAs that can regulate the expression of tumour suppressor genes [14,16]. Moreover, TEX play a major role in evading the anti-tumour immune response by triggering an immunosuppressive microenvironment [17,19]. Exosomes are also able to inhibit the function of immune cells involved in the anti-tumour response (dendritic cells, natural killer, CD4^+^ and CD8^+^ T lymphocytes) and to activate regulatory cell populations with immunosuppressive activities (Tregs and Bregs).

Infection with oncogenic herpesviruses, such as Epstein–Barr herpesvirus (EBV) (human gammaherpesvirus 4) and Kaposi’s sarcoma-associated herpesvirus (KSHV) (human gammaherpesvirus 8), can alter the content of exosomes released by infected cells, thereby leading to increased viral propagation, promotion of virus-induced tumourigenesis and modulation of the tumour microenvironment [20,21]. Exosomes derived from EBV-latently infected B-cells have been shown to confer strong proliferative and differentiation capacities to recipient cells [22,23]. The EBV oncoprotein LMP-1 transferred via exosomes plays a major role in promoting B-cell proliferation by mimicking CD40 signalling and activating MAPK/ERK and PI3K/Akt signalling pathways through paracrine or autocrine mechanisms in endothelial cells [21,23]. Additionally, the release of LMP-1 leads to increased expression of ICAM-1 and EGFR in recipient cells, two factors that are strongly linked to tumourigenic processes [21,24]. At the same time, exosomes derived from EBV-transformed cells can also exert immunosuppressive activities [25]. Among the viral factors carried by these EVs, the LMP-1 protein seems to exert an inhibitory effect on the proliferation and activation of T lymphocytes [26]. Cellular proteins and viral non-coding RNAs (miRNAs and EBV-encoded small RNAs (EBERs)) enriched in exosomes from EBV-transformed cells also contribute to the establishment of an immunosuppressive microenvironment, notably by inhibiting apoptosis [26,30]. In contrast to EBV, EVs released by KSHV-infected cells do not contain viral proteins but carry cellular biomolecules (proteins and mitochondrial DNA) that modulate the immune response [20,31, 32]. Cellular proteins and miRNAs present in EVs released upon KSHV infection are also largely involved in the reprogramming of recipient cells, particularly endothelial cells, thereby promoting KSHV-induced tumourigenesis [33,34].

To date, the role of EVs in the lifecycle of avian herpesviruses is only poorly understood. Molecular characterization of exosomes isolated from the sera of tumour-bearing chickens (TEX), and more recently from an MDV-transformed cell line, has revealed the presence of viral mRNAs, miRNAs and proteins enriched in EVs that may play important roles in the regulation of the immune response and virus-induced tumourigenesis. However, the mechanisms involved in these processes are yet to be elucidated [35,36].

In the present study, we set out to examine whether EVs released by MDV-transformed T lymphocytes can influence the proliferation of chicken immune cells and aimed to identify viral and/or cellular molecules contained in the vesicles that may contribute to this process. Altogether, we trust that our findings provide fresh new insights into the contribution of EVs to tumour progression and the mechanisms driving Marek’s disease pathogenesis.

## Methods

### Cells

The 3867 K-lymphoblastoid cell line was derived from a lymphoma induced in the kidneys of a chicken infected with the highly pathogenic recombinant virus vRB-1B 47EGFP MDV [37]. Cells were grown in suspension in RPMI 1640 supplemented with 2 mM glutamine, 10% tryptose phosphate broth, 4.5 g l^−1^ glucose, 1% sodium pyruvate, 1% non-essential amino acids and 10% FBS. The chicken endothelial cell line (chAEC) established from primary aortic endothelial cells [38,39] was cultured in complete macrovascular Endothelial Cell Growth Medium (EGM-2 BulletKit, Lonza). The chicken macrophage cell line HD11 transformed by avian myelocytomatosis type MC29 virus was grown at 37 °C with 5% CO2 in DMEM supplemented with 2 mM glutamine, 1% sodium pyruvate and 10% FBS [40]. Chicken embryo skin cells (CESCs) were prepared from 12-day-old specific-pathogen-free (SPF) embryos of White Leghorn layers and maintained in culture as previously described [41].

Chicken primary B- and T-cells were isolated from 6-week-old SPF White Leghorn chickens (B13/B13 haplotype) provided by the INRAE PFIE animal experimental platform (Plate-forme d’Infectiologie Expérimentale, INRAE Centre Val de Loire, Nouzilly, France). Monocyte cells were prepared from the bursa of Fabricius and spleens by dissociation of the organs and isolation of cells by density gradient centrifugation on Ficoll-based lymphocyte separation medium (Eurobio), as previously described [42]. Monocytes were cultured in Iscove’s modified Dulbecco’s medium (Gibco, Fisher Scientific), 100 U ml^−1^ penicillin, 100 μg ml^−1^ streptomycin, 8% (vol/vol) FBS and 2% (vol/vol) chicken serum at a density of 2×10^5^ cells in 96-well plaques. B-cells were activated for 4 h using recombinant soluble chicken CD40 ligand (chCD40L) serum [43]. T-cell activation was induced by culturing cells in tissue culture dishes coated overnight at 4 °C with 1 μg ml^−1^ of the TCR-2 monoclonal antibody (recognizing the *α*V*β*1 T-cells) [44]. Both chCD40L serum and TCR-2 antibody were kindly provided by Dr. Sonja Haertle (LMU Munich, Germany).

All cells were maintained at 41 °C in a 5% CO_2_ atmosphere.

### Isolation of EVs

The 3867 K cells were cultured for 48 h in complete medium and then washed twice in PBS and further cultured for 48 h in complete medium depleted of contaminating vesicles. This medium was obtained by overnight ultracentrifugation of RPMI 1640 supplemented with 10% FBS at 110,000 ***g*** in a 45Ti rotor at 4 °C. EVs released by 3867K were collected using differential ultracentrifugations. Cells grown in EV-depleted medium were spun down by centrifugation at 1,000 ***g*** for 7 min. The supernatant was transferred to a new tube and centrifuged at 2,000 ***g*** for 20 min at 4 °C to pellet apoptotic bodies and cellular debris. The supernatant was then centrifuged for 30 min at 10,000 ***g*** at 4 °C in a rotor swing SW28 to pellet the MV-enriched fraction (also called 10K-EVs). Finally, the EXO-enriched fraction (100K-EVs) was recovered from the pellet resulting from the last centrifugation step (70 min at 100,000 ***g*** at 4 °C). Pellets corresponding to cells, 10K- and 100K-EVs were washed in PBS, centrifuged within the same conditions used for their isolation and resuspended in 50–100 µl of sterile PBS. Protein concentration was estimated using the Micro BCA Kit (Thermo Scientific) according to manufacturer instructions. Concentration was adjusted to 1 µg µl^−1^ either in culture media or in Laemmli 2 × sample buffer. Of note, to ensure that EV preparations were not contaminated by EVs and/or serum-derived components from the conditioned medium, which could bias subsequent biological experiments, the medium alone underwent the same successive ultracentrifugation steps as those used for EV isolation. Protein levels in the medium were below the detection threshold of the Micro BCA protein assay kit (Fig. S1a, available in the online Supplementary Material). Moreover, splenocyte exposure to the medium alone failed to induce proliferative responses, confirming that the biological effects observed with EVs are not attributable to the medium itself (Fig. S1b).

All relevant data of our experiments were submitted to the EV-TRACK knowledge base (EV-TRACK ID: EV250100) [45].

### Transmission Electron microscopy (TEM)

The 10K- and 100K-EVs preparations were deposited onto carbon-coated copper grids for 1 min before being washed with distilled water. The negative staining was then performed for 1 min with a solution of 2% uranyl acetate in distilled water. After being dried thoroughly, the grids were observed with a transmission electron microscope JEOL 1011 (Jeol). Images were acquired using a Rio9 camera (Gatan) and processed with Digital Micrograph V.3.42.3048.0 software (Gatan).

### Antibodies and immunoassays

Five micrograms of cellular and EV proteins extracted in 2× Laemmli buffer were separated on a 10% SDS-PAGE. Proteins were then transferred to a nitrocellulose membrane using the Trans-Blot Turbo Transfer System (Bio-Rad), blocked in Tris 10 mM pH 8.25, NaCl 150 mM, Tween 0.2% containing milk 3% and subsequently incubated with the mouse monoclonal anti-GAPDH (MAB374, Chemicon), anti-*α*-tubulin (T9026, Sigma-Aldrich), anti-actin (A4700, Sigma-Aldrich), anti-MHC class I (AM08130PU-N, Origene), anti-MHC class II (AM08131PU-N, Origene), anti-flotillin-1 (#610820, BD Biosciences), anti-Hsc-70 (#610607, BD Biosciences), anti-Rab7 (Sc-376362, Santa Cruz), anti-CD63 (Sc-5275, Santa Cruz) or the rabbit polyclonal anti-Tsg101 (A303-507A-T, Bethyl Laboratories) primary antibody. Goat anti-mouse IgG (#A4416, Sigma-Aldrich) or goat anti-rabbit IgG (#A0545, Sigma-Aldrich) conjugated to peroxidase were used as secondary antibodies. Peroxidase signals were revealed using Immobilon Western Chemiluminescent HRP substrate (P90720, Millipore). The chemiluminescence signal was acquired using a Fusion FX7 system.

### Visualization of 100K-EVs internalization by fluorescence microscopy

100K-EVs were labelled using the PKH67 Green Fluorescent Cell Linker Kit according to the manufacturer’s instructions (Sigma-Aldrich). Briefly, the 100K-EVs pellet isolated from a culture of about 3×10^8^ cells was resuspended in 1 ml of diluent C, and one volume of 2 × dye solution containing 4×10^−6^M of ethanolic PKH67 was added. After 4 min of continuous mixing by pipetting at room temperature (RT), the staining was quenched by adding 2 ml of EV-depleted FBS (System Biosciences). The labelled-100K-EVs were then washed twice in PBS with an ultracentrifugation step of 60 min at 100,000 ***g*** at 4 °C between each wash, and the pellet was resuspended in 1 ml of RPMI 1640. EV-free PBS treated with the same staining procedure was used as a negative control. CESCs and chAEC recipient cells grown onto coverslips in 24-well plates were rinsed twice in serum-free medium before the addition of 250 µl of labelled 100K-EVs (or EV-free PBS). After 30 min, 60 min and 120 min of incubation at 41 °C, cells were washed three times in PBS and fixed with paraformaldehyde 4% for 20 min at RT. To visualize intracellular structures, cells were permeabilized with 0.5% Triton X-100 for 5 min at RT and blocked with PBS, 0.1% Triton X-100, 2% bovine Serum Albumin. The cytoskeleton was visualized by labelling either actin filaments or tubulin structures. Actin filaments were stained using phalloidin conjugated to Alexa Fluor 594 (Invitrogen), following the manufacturer’s protocol. Alternatively, tubulin structures were revealed using a monoclonal anti-tubulin antibody (Sigma-Aldrich; 1 : 500 dilution), followed by a secondary goat anti-mouse IgG antibody conjugated to Alexa Fluor 594 (Invitrogen). Cell nuclei were counterstained with Hoechst 33342 dye (Invitrogen). Internalized 100K-EVs labelled with PKH67 Green Fluorescent were directly observed. Cells were observed under an Axiovert 200 M inverted epifluorescence microscope equipped with a 63’ Plan Apochromat oil/DIC and an Apotome imaging system (Zeiss). Images were captured with an AxioCam MRm charge-coupled-device camera (Zeiss) using AxioVision software.

### Lipidomic analysis

100K-EVs and 10K-EVs were collected from supernatants of 3867K and sonicated for 1 min. From three 100K-EVs and 10K-EVs biological replicates, lipids were resuspended in 10 µL of 2,5-dihydroxyacetophenone matrix at 20 mg ml^−1^ dissolved in 90% methanol/9.8% ultrapure H_2_O in the presence of 0.2% TFA. The matrix/sample mix (1:1.5 µl; v/v) was deposited onto a ground steel 384 MALDI plate (Bruker Daltonics, Bremen, Germany) using the dried droplet method and allowed to evaporate at room temperature for 30 min. For each sample, 10 spots (technical replicates) were prepared, and spectra were manually acquired by MALDI-TOF MS. Mass spectrometric analyses were performed on an UltrafleXtreme MALDI-TOF-TOF mass spectrometer (Bruker Daltonics, Bremen, Germany), controlled by FlexControl 3.0 software (Bruker Daltonics, Bremen, Germany), operating in reflectron geometry and equipped with a 355 nm Nd:YAG Smartbeam II™ laser at a 2 kHz laser repetition rate. Spectra were obtained in positive ion mode in the 100–1200 m/z range and collected from each spot as a sum of 1,000 laser shots in five shot steps (total of 5,000 spectra per spot). The parameters used for spectra acquisition were ion source 1,+25.23 kV; ion source 2,+22.45 kV; lens, +8.01 kV; pulsed ion extraction at 120 ns; and laser parameter set at medium. External calibration using a quadratic calibrant algorithm was performed using a ‘homemade’ calibrant solution (1 µl of calibrant solution plus 1 µl of matrix) containing caffeine, MRFA peptide, bradykinin 2–9, bradykinin, angiotensin I and Glu1-fibrinopeptide B. To increase mass accuracy, internal calibration was performed. A lock mass correction using flexAnalysis 4.0 software (Bruker Daltonics, Bremen, Germany) was achieved with the mass corresponding to the phosphatidylcholine 34:1 (PC 34:1; [M+H]+ : 760.5780 m/z). Spectral processing and analysis were performed with ClinProTools v3.0 software (Bruker Daltonics, Bremen, Germany). Data analysis began with an automated raw data pre-treatment workflow, comprising baseline subtraction (top hat, 10% minimum baseline width), three smoothing at 0.2 m/z using the Savitzky–Golay algorithm. Spectra realignment was performed using prominent peaks (maximal peak shift 1,000 p.p.m., 30% of peaks matching most prominent peaks). Normalization on peak intensity was performed using the total ionic count in order to display and compare all spectra on the same scale. Automatic peak detection was applied to the total average spectrum with a signal/background noise greater than 2. For differential analyses, MALDI spectra (*N*=30 per dataset 100K-EVs or 10K-EVs) were compared using the non-parametric Wilcoxon test. The repeatability for 100K-EVs or 10K-EVs analyses linked directly to the spectrometer process or to the biological replicates (*N*=3) was evaluated by a coefficient of variation (CV). For 348 detected peaks, mean CV values did not exceed 29.40% and 23.78%, respectively, for 100K-EVs and 10K-EVs. Masses were considered statistically differential between groups if the *P*-value was <0.01 with a fold change ratio >1.5. Hierarchical clustering was performed using the gplots (v3.0.3) and FactoMineR (v2.1) packages of the R software.

### Preparation of EV protein samples for proteomic analysis: in-gel digestion

Proteins included in 10K and 100K fractions were resuspended in Laemmli buffer, and 100 µg of proteins was separated by a 4–20% SDS-PAGE, followed by Coomassie Brilliant Blue G250 staining (GeLC-MS/MS analysis). The SDS-PAGE gel was cut into 10 sections, and each slice was rinsed separately in water and then in acetonitrile solution (1:1, 5 min), followed by 100% acetonitrile (10 min). Reduction and cysteine alkylation were performed by successive incubation with 10 mM dithiothreitol in 50 mM NH_4_HCO_3_ (30 min, 56 °C) and then 55 mM iodoacetamide in 50 mM NH_4_HCO_3_ (20 min, RT, in dark). Gel pieces were then incubated with 50 mM NH_4_HCO_3_ and acetonitrile (1:1, 10 min), followed by acetonitrile (15 min). Proteolytic digestion was carried out overnight using 25 mM NH_4_HCO_3_ with 12.5 ng µl^−1^ trypsin (Roche Diagnostics). Peptides were then extracted by incubation in 5% formic acid (sonicated) with the supernatant removed and saved, followed by incubation in acetonitrile and 1% formic acid (1:1, 10 min) and a final incubation with acetonitrile (5 min); again, supernatant was removed and saved. These two peptide extractions were pooled and dried using a SPD1010 speedvac system (Thermosavant, Thermo Fisher Scientific), and the resultant peptide mixture was analysed by nanoflow liquid chromatography-MS/MS (nanoLC-MS/MS).

### NanoLC-MS/MS

After in-gel digestion, peptide mixtures from each gel band were analysed in triplicate by nanoLC-MS/MS. All experiments were performed on a dual linear ion trap Fourier transform mass spectrometer, the LTQ Orbitrap Velos (Thermo Fisher Scientific), coupled to an Ultimate® 3000 RSLC ultra-high-pressure liquid chromatographer (Thermo Fisher Scientific) controlled by Chromeleon Software (version 6.8 SR11, Dionex). Samples were desalted and concentrated on an LCPackings trap column (Acclaim PepMap 100 C18, 75 µm inner diameter × 2 cm long, 3 µm particles, 100 Å pores) for 10 min at 5 µl min^−1^ with 4% solvent B (15.9% water and 84% acetonitrile in the presence of 0.1% formic acid) in solvent A (97.9% water and 2% acetonitrile in the presence of 0.1% formic acid). The peptide separation was conducted using a LCPackings nano-column (Acclaim PepMap C18, 75 µm inner diameter × 50 cm long, 2 µm particles, 100 Å pores) at 300 nL min^−1^ by applying a gradient of 4–60% of solvent B for 90 min. Data were acquired in positive mode in data-dependent mode to automatically switch between high-resolution full-scan MS spectra (R=60,000) collected in profile mode and low-resolution CID-MS/MS in centroid mode (m/z 300–1800). The 20 most intense peptide ions with charge states ≥2 were sequentially isolated and fragmented in the high-pressure linear ion trap by low-energy CID (collision energy 35%, activation time 10 ms, Qz 0.25). Dynamic exclusion was activated for 30 s with a repeat count of 1.

### Protein identification and data validation

MS/MS ion searches were performed using Mascot search engine version 2.3.2 (Matrix Science, London, UK) via Proteome Discoverer 2.1 software (Thermo Fisher Scientific) against the chordata and viruses sections of the National Center for Biotechnology Information (NCBI) non-redundant database. The search parameters included trypsin as a protease with two allowed missed cleavages and carbamidomethylcysteine, methionine oxidation and acetylation of N-terminal protein as variable modifications. The tolerance of the ions was set to 5 p.p.m. for parent and 0.8 Da for fragment ion matches. Peptides and proteins identified by MASCOT were validated using the Peptide and ProteinProphet algorithms with Scaffold software (version 4.8.2, Proteome Software). Protein identifications were accepted if they contained at least two identified peptides. Keratins were not taken into consideration for further analysis since they could only be derived from skin contaminations. The abundance of identified proteins was estimated by calculating the exponentially modified protein abundance index (emPAI) using Scaffold Q+software (version 4.3, Proteome Software, Portland, USA).

### Functional annotation using gene ontology

Gene Ontology (GO) term annotations for the biological processes and cellular components categories provided by the GO consortium (http://www.geneontology.org/) were investigated using a protein database with UniprotKB (http://www.uniprot.org) and genomic databases with Ensembl (http://www.ensembl.org) and NCBI (https://www.ncbi.nlm.nih.gov).

### GO analyses

Proteins identified by MS were categorized and clustered according to the ‘biological processes’ and ‘signalling pathways’ in which they are involved using the PANTHER (Protein Analysis Through Evolutionary Relationships, http://pantherdb.org) and the Kyoto Encyclopedia of Genes and Genomes (KEGG) databases, respectively.

### Cell proliferation–bromodeoxyuridine assay

Monocytes isolated from spleen, thymus and bursa were plated at a density of 2×10^5^ cells per 96-well. Cells were either activated for 1 h at 41 °C using recombinant soluble chCD40L serum to enrich the B-cell population or by culturing cells in tissue culture dishes coated with the TCR-2 monoclonal antibody to enrich the T-cell population. Non-stimulated cells were grown without chCD40L and anti-TCR-2, as a control. EVs (100K or 10K) were then added to cell cultures at concentrations corresponding to 1 µg, 2 µg, 4 µg or 8 µg of vesicular protein content. Cell proliferation was assessed using the ELISA BrdU colourimetric assay (Roche Life Science). The bromodeoxyuridine (BrdU) labelling solution (1 : 1000) was added 6 h after adding the EVs to the culture medium, and the cells were incubated for an additional 16 h prior to proceeding with the protocol recommended by the manufacturer. The absorbance of the samples was measured in an ELISA reader (Multiskan Ascent, Thermo Labsystem) at 370 nm. Results obtained from stimulated cells were normalized to those from non-activated cells (cultured without chCD40L or anti-TCR-2) and are expressed as means (±sd) from three independent experiments, each one done in triplicate.

### Flow cytometry analyses

Splenocytes activated with anti-TCR2 (2.10^5^ cells/P96 well) were cultured for 24 h in the presence of 8 µg of 100K-EVs or in medium without EVs as a negative control. Cells were then washed once in PBS and incubated with mouse anti-chicken CD4-PE (#8210-09, Southern Biotech) or CD8-FITC antibodies (#8220-02, Southern Biotech). Cells were detected by flow cytometry using a MoFlo Astrios EQ high-speed cell sorter (Beckman Coulter, Inc.), and data were analysed using the FlowJo software (FlowJo LLC). Viable cells were gated on morphological criteria (FSC/SSC) in order to eliminate cellular debris and damaged cells. The CD4 and CD8 positive cells were then detected on the viable population.

### Statistical analyses

All graphs and statistical analyses were performed using the GraphPad Prism software version 5.02. Data are presented as means and standard deviations (±sd) or medians. A Mann–Whitney (two-tailed) test was used to compare non-parametric variables between two groups. *P*-values<0.05 were considered statistically significant as indicated in the figure legends.

## Results

### Characterization of two distinct types of vesicles from MDV-transformed T-cells

**Fig. 1.**
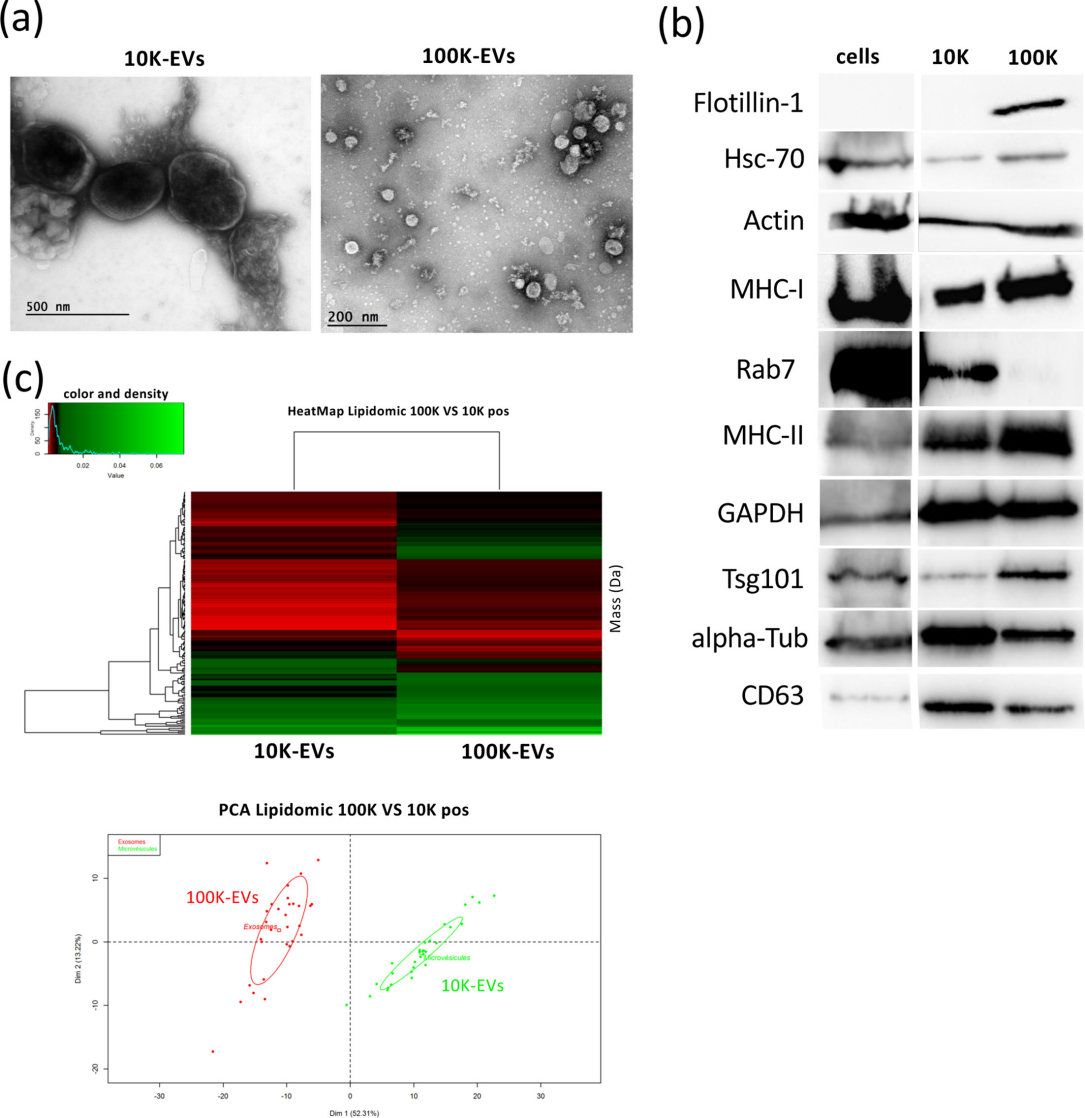
Characterization of EVs released by MDV latently-infected cells. 10K-EVs and 100K-EVs were isolated by differential ultracentrifugations from supernatants of the 3867K MDV-transformed T-cell line. (a) Representative images of 10K-EVs (>100 nm) and 100K-EVs (30–100 nm) in EV preparations observed by transmission electron microscopy. (b) Immunoblot analysis of EVs for canonical EV protein markers. (c) Qualitative analysis of lipids composing the 10K and 100K vesicles. Results are shown as a heat map of the average spectral profiles of three biological replicates, and a two-dimensional principal component analysis was performed to compare 100K and 10K vesicles.

EVs were isolated from the supernatant of the MDV-transformed T-cell line 3867K using differential ultracentrifugations. Transmission electron microscopy analysis of the 10K and 100K-EVs fractions revealed that EVs included in the 10K fraction were polymorphic with a size ranging from 200 to 1,000 nm, whereas EVs in the 100K fraction were homogeneous with a size between 30 and 100 nm (Fig. 1a). To further characterize these EVs, immunoblots were performed using proteins extracted from the 3867 K cells, as well as the 10K and 100K pellets (Fig. 1b). Canonical EV markers were readily detected, including membrane proteins (tetraspanin CD63 and flotillin 1), cytosolic proteins (rab7, tumour-susceptibility protein, TSG101), heat shock protein Hsc-70 and MHC molecules. More specifically, small GTPase Rab7, alpha-tubulin and CD63 were enriched in 10K vesicles, while flotillin-1, MHC-II and TSG101 were enriched in 100K vesicles. Additionally, we demonstrated by a lipidomic approach that the 10K- and 100K-EVs differ in their lipid composition (Fig. 1c), indicating that they originate from distinct cellular compartments and could respectively be associated with MVs and EXOs.

### Protein content of 10K- and 100K-EVs isolated from MDV-transformed T-cells

**Fig. 2.**
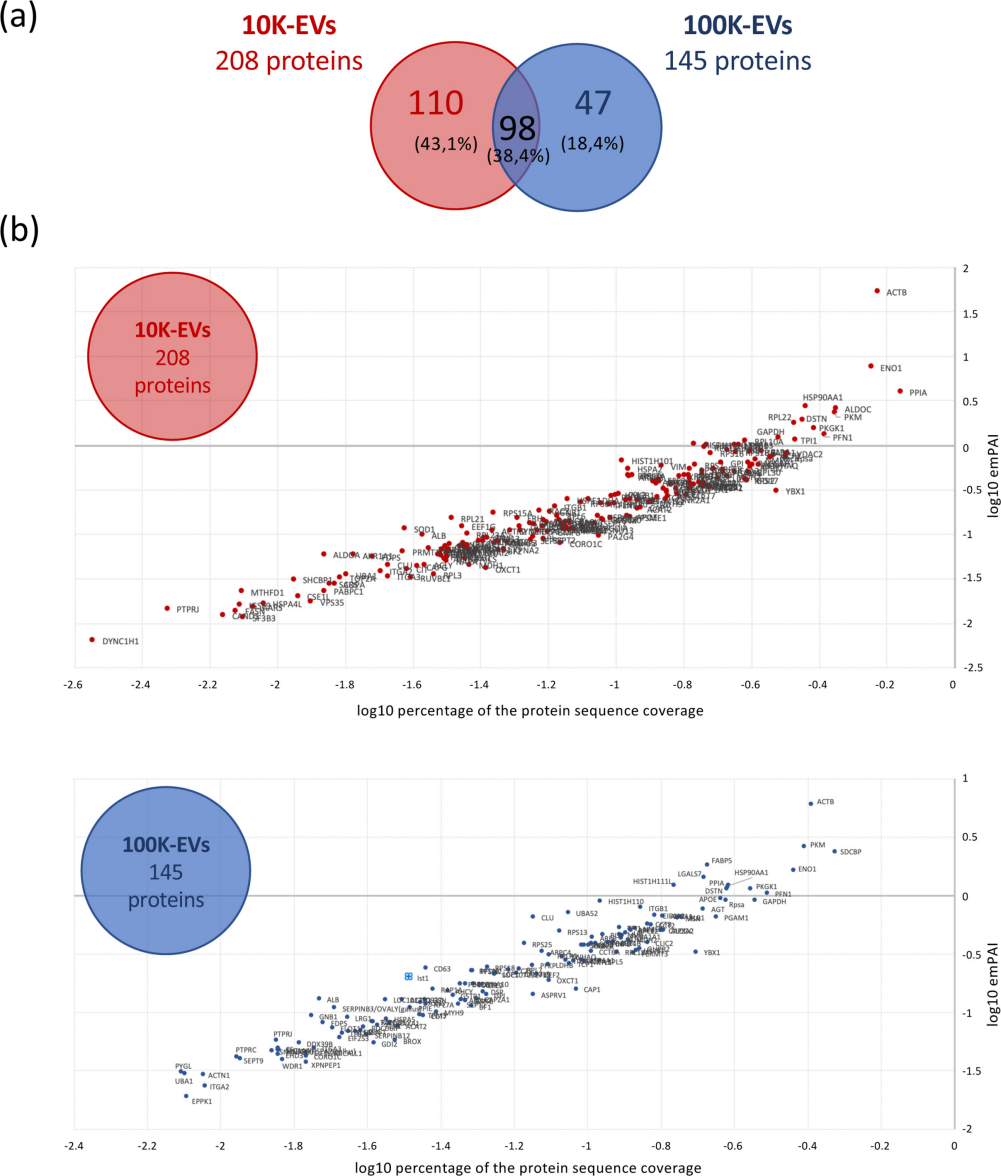
Protein distribution in 10K-EVs and 100K-EVs isolated from 3867K. The protein composition of 10K-EVs and 100K-EVs isolated from the 3867 K cell line was analysed by nanoLC-HR-MS/MS. (**a**) Venn diagram showing the distribution of the 255 different proteins identified exclusively in 10K-EVs, in 100K-EVs or in both types of EVs. (**b**) Quantitative distribution of the proteins identified by nanoLC-MS/MS based on the protein sequence coverage and emPAI.

Proteomic analysis of the 10K-EVs and 100K-EVs derived from 3867 K cells was conducted using nanoLC-HR-MS/MS. A total of 208 and 145 proteins were identified in 10K-EVs and 100K-EVs, respectively (Fig. 2a). Among the 255 distinct proteins identified in EVs released from 3867 K cells, 98 were common to both vesicle types, 110 were unique to 10K-EVs and 47 were specific to 100K-EVs (Tables S1 and S2). Strikingly, we did not detect any MDV proteins in EVs released from 3867 K cells. The proteomics data confirmed the presence of proteins typically found in mammalian 10K-EVs (MVs) and 100K-EVs (EXOs) (e.g. Actin, GAPDH, MHC-I and II, CD63) and proteins specifically enriched in 10K-EVs (e.g. HSC-70 and actinin-4) or in 100K-EVs (e.g. flotillin-1, *α*4 and *β*-1 integrins, Alix, Tsg101).

**Fig. 3.**
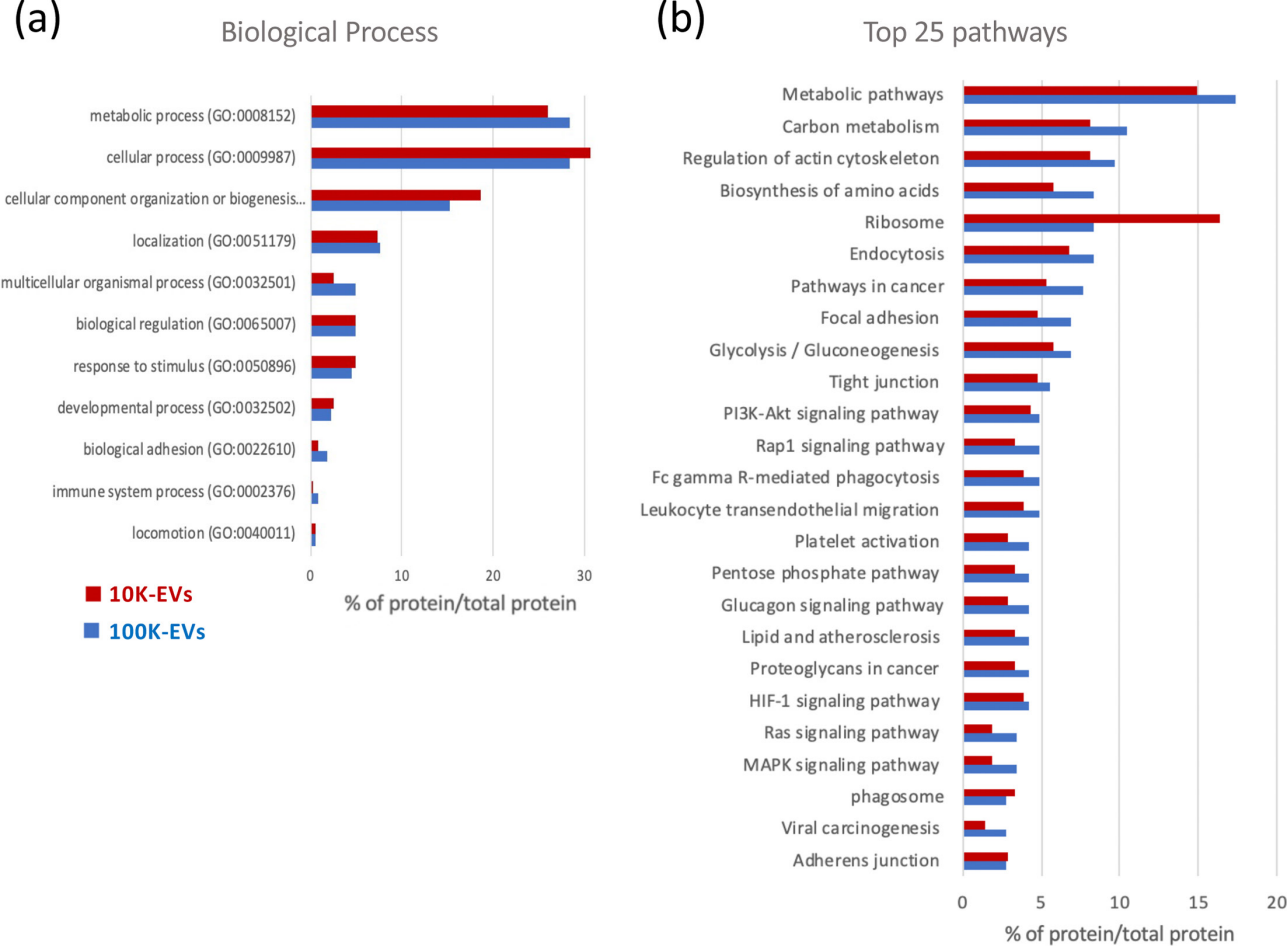
Functional annotation of vesicular proteins. GO functional annotation of EV protein composition according to biological process (**a**) and signalling pathways (**b**) categories. The results are shown as a percentage of protein involved in the considered process/pathway, reported to the total protein identified in EVs. Results related to 10K-EVs and 100K-EVs are shown in red and blue, respectively.

To identify the most enriched proteins in each vesicle type, absolute protein quantification was performed by plotting the Log10 of the emPAI for each protein against the Log10 of its sequence coverage percentage (Fig. 2b, Table S3). The ten most abundant proteins identified in 10K-EVs and 100K-EVs are listed in Table 1. Collectively, these proteins represent 59.8% and 35.61% of the total proteins identified in the 10K-EVs and 100K-EVs, respectively. The beta-actin (ACTB) protein is the most abundant in both types of vesicles, representing 40% of total proteins in 10K-EVs and 10.4% in 100K-EVs. The RPL22 protein was exclusively found in 10K-EVs, whereas SDCBP, FABP5 and LGALS7 were specifically enriched in 100K-EVs. These proteins are likely to modulate key pathways in recipient cells including glycolysis (ENO1, ALDOC, PKM or PKGK1), host–virus interactions (PPIA and HSP90AA1), cell death and proliferation (SDCBP, LGALS7 and PPIA) and actin dynamics (ACT B, DSTN and PFN1). Additionally, relative protein quantification based on spectral counting analysis revealed significant differences in protein abundance between the two vesicle types (Table 2). Proteins such as RAP1A, ACAT2, RPL10A and ACTN1 were found to be 10- to 15-fold more abundant in 10K-EVs compared to 100K-EVs, whereas other proteins (e.g. CLU, APOE, CLTC and LPL) were predominantly enriched in 100K-EVs. To more precisely determine the biological functions and molecular interaction networks associated with proteins enriched in 10K-EVs and 100K-EVs, we then performed GO and KEGG pathway analyses. The GO functional annotation evidenced that the proteins contained in both vesicle types may be involved in similar biological processes, including cellular metabolism (such as glycogenesis and regulation of gene expression), cellular functions (e.g. actin cytoskeleton organization, cell cycle, cell death, protein folding and exocytosis), biogenesis and organization of cellular components and localization of cellular proteins. To a lesser extent, they may also play a role in locomotion/migration, adhesion and regulation of the immune system (Fig. 3a). The KEGG pathway analysis further confirmed that proteins found in 10K-EVs and 100K-EVs might influence similar pathways, particularly those related to metabolism and cytoskeleton regulation (Fig. 3b). Interestingly, this analysis also revealed that a non-negligible number of proteins present in these vesicles could participate in oncogenic pathways (pathways in cancer, focal adhesion, PI3K-Akt, proteoglycans in cancer, HIF-1, MAPK signalling pathways, viral carcinogenesis and adherens junction).

**Table 1.** Top ten abundant proteins found in 10K- or 100K-EVs using GeLC-MS/MS analysis

Gene symbol	Gene description	10K-EVs		100K-EVs	
		% emPAI	Rank	% emPAI	Rank
ACTB	Beta-actin	40.23	1	10.41	1
ENO1	Alpha-enolase	5.73	2	2.82	5
PPIA	Peptidyl-prolyl cis-trans isomerase A	3.04	3	1.98	10
HSP90AA1	Heat shock protein HSP 90-alpha	2.05	5	2.09	7
ALDOC	Fructose-bisphosphate aldolase C	1.95	4	0.87	*
PKM	Pyruvate kinase	1.79	6	4.53	2
DSTN	Destrin	1.46	7	2.02	9
RPL22	60S ribosomal protein L22	1.36	8	0	*
PKGK1	Phosphoglycerate kinase	1.18	9	1.957	*
PFN1	Profilin-1-like	1.01	10	1.809	*
HIST1H111L	Histone H1.11L	0.75	*	2.09	8
SDCBP	Syntenin-1	0	*	4.07	3
FABP5	Fatty acid-binding protein, epidermal	0	*	3.14	4
LGALS7	Galectin-7	0	*	2.46	6

Absolute quantitative values are expressed as the percentage of the emPAI (% emPAI). (*) indicates that the corresponding proteins are not among the top ten abundant proteins in the considered vesicles.

**Table 2.** Differentially enriched proteins in 10K-EVs vs. 100K-EVs

Gene symbol	Gene description	10K/100K-EVs	
		*P*-value	FC (SC)
RAP1A	Rap1A-retro1	0.049	15
ACAT2	Acetyl-CoA acetyltransferase, cytosolic	0.026	13
RPL10A	60S ribosomal protein L10a	0.0049	11
ACTN1	Alpha-actinin	0.048	11
RPL7A	60S ribosomal protein L7a	0.004	9.6
GAPDH	Glyceraldehyde-3-phosphate dehydrogenase	0.01	8.1
ACTB	Beta-actin	0.02	7.4
GPI	Glucose-6-phosphate isomerase	0.0026	7.2
GDI2	Rab GDP dissociation inhibitor beta	0.014	7.1
ALDOC	Fructose-bisphosphate aldolase C	0.0041	6.9
*CCT3*	*T-complex protein 1 subunit gamma*	*0.0093*	*0.6*
*CCT6A*	*T-complex protein 1 subunit zeta*	*<0.00010*	*0.3*
*ITGB1*	*Integrin beta-1 precursor*	*0.0027*	*0.3*
*LPL*	*Lipoprotein lipase precursor*	*0.0086*	*0.2*
*CLTC*	*Clathrin heavy chain 1*	*0.00062*	*0.1*
*APOE*	*Apolipoprotein E*	*0.029*	*0.1*
*CLU*	*Clusterin preproprotein*	*0.0043*	*0.03*

Relative quantitative values are expressed as 10K/100K-EVs fold change (FC) calculated from spectral counting (SC) values. *P*-values (<0.05) are indicated. The ten proteins most enriched in 10K-EVs compared to 100K-EVs are presented in regular font, whereas proteins depleted in 10K-EVs (and therefore enriched in 100K-EVs) are shown in italics.

### The 100K-EVs from MDV-transformed cells are internalized by chicken cells

**Fig. 4.**
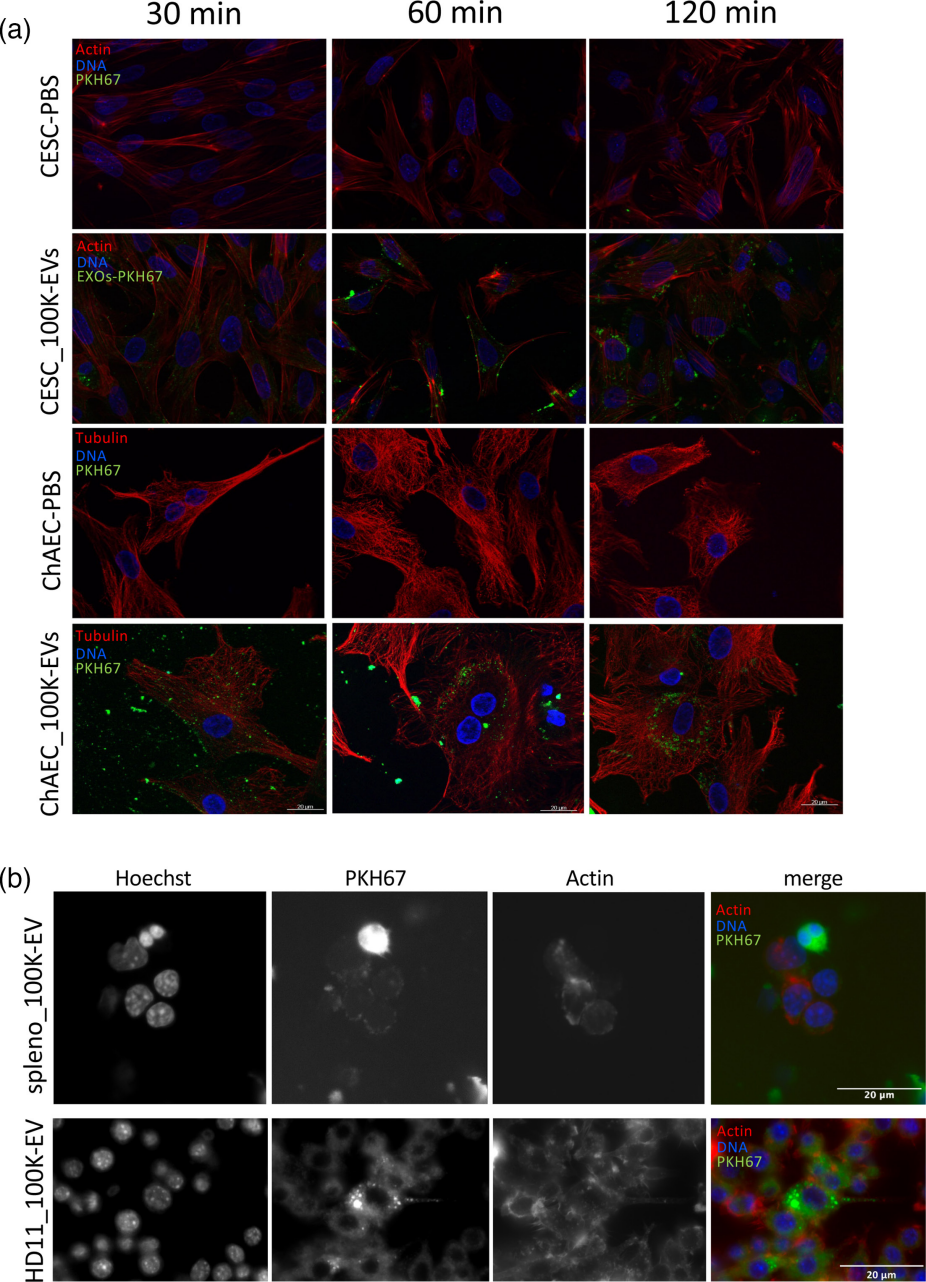
Internalization of 3867K-EVs by recipient avian cells. The 100K-EVs isolated from 3867K cultures were labelled with the green fluorescence dye PKH-67. As a negative control, PBS underwent the same staining procedure. The labelled EVs were then incubated with two sets of recipient cells: (**a**) embryonic skin cells (CESCs) and aortic endothelial cells (chAECs) and (**b**) immune cells (splenocytes and HD11 macrophages). At indicated time points for CESCs and chAECs, and 4 h post-contact for splenocytes and HD11 cells, cells were fixed and stained for actin or tubulin (red). Nuclei were counterstained with Hoechst 33342 (blue) and cellular uptake of EVs was monitored by fluorescence microscopy.

Prior to assessing the potential activity of EVs on cells, we verified that EVs released by 3867K could be internalized by recipient cells. The 100K-EVs isolated from 3867K cultures were labelled with the PKH67 dye (green fluorescence) and added to two MDV permissive cell types, CESCs or chAECs, for 30, 60 and 120 min before being monitored by fluorescent microscopy (Fig. 4a). As early as 30 min post-contact, 100K-EVs were observed at the cell surface and began to be internalized by both types of recipient cells. By 60 min, they appeared as punctate intracytoplasmic staining dots progressively moving toward the nucleus over time, confirming their uptake via the endocytic pathway. Likewise, we observed the internalization of 100K-EVs in avian immune cells, splenocytes and the HD11 macrophage cell line (Fig. 4b).

### EVs produced by MDV-transformed cells modulate the proliferation of immune cells

**Fig. 5.**
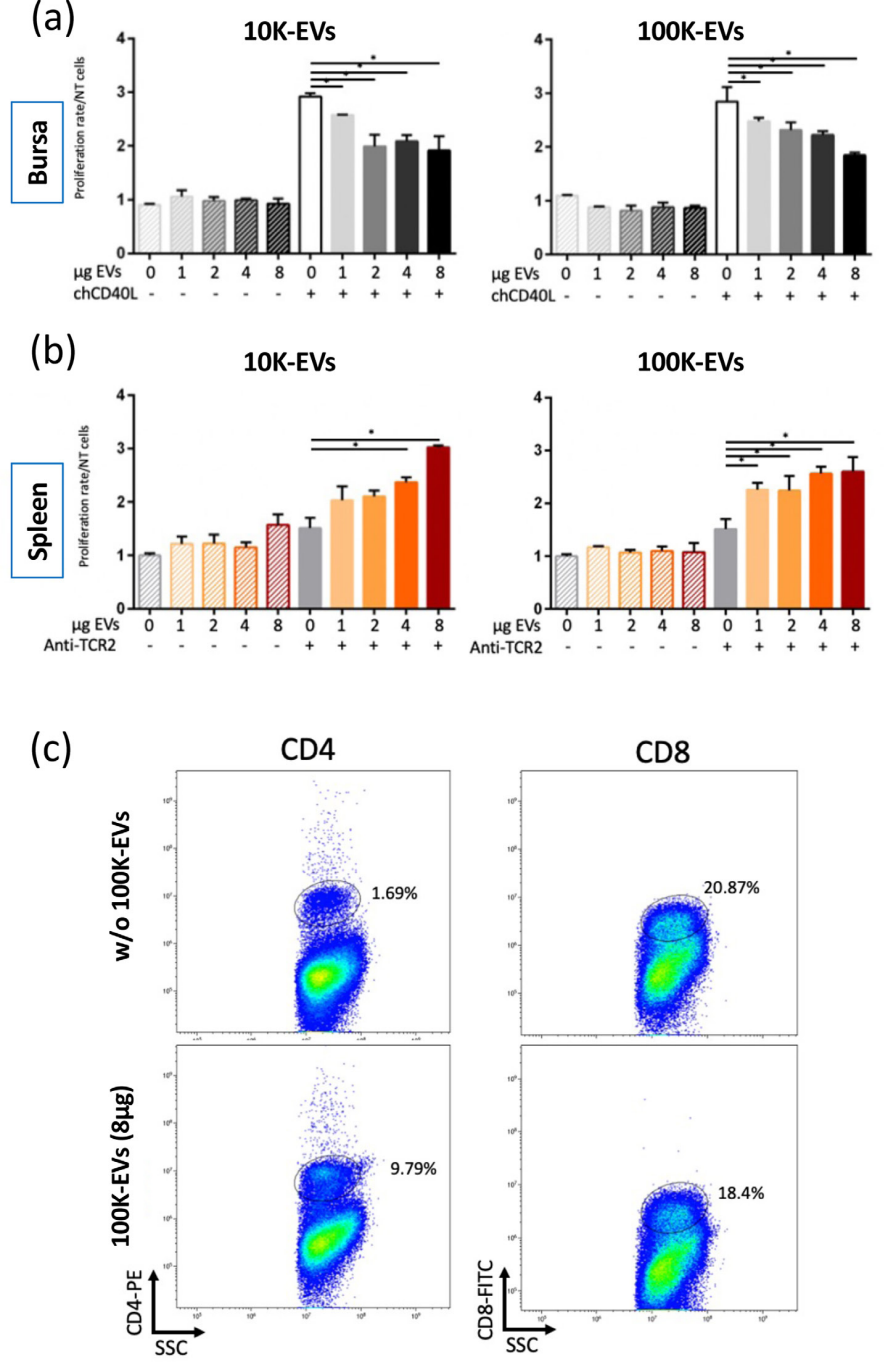
Impact of 3867K-EVs on B- and T-cell proliferation. Monocytes were isolated from the bursa of Fabricius and spleens by ficoll-gradient purification following mechanical dissociation of the organs. B-cell populations were enriched through activation of bursal monocytes with chCD40L, while T-cell populations were derived from isolated splenocytes and stimulated using the TCR-2 monoclonal antibody. The 10K-EVs or 100K-EVs were added at different concentrations on activated cells (and non-activated cells, as a control). Cell proliferation was measured by BrdU assay at 24 h post-culture with EVs. The proliferation of chCD40L-activated bursal cells (**a**) or TCR2-activated splenocytes (**b**) cultured in the presence of EVs was related to that of cells cultured in the absence of EVs. Results from three independent experiments are presented as means±sd. *P<0.05. (**c**) TCR2-activated splenocytes were cultured for 24 h with 8 µg of 100K-EVs (or without, as a negative control). The expression of CD4 or CD8 T-cell markers was assessed by immunostaining and flow cytometry. Representative dot plot diagrams from two independent experiments are presented with the percentage of CD4 and CD8 positive cells within the viable cell population.

Our proteomic analysis suggests that proteins contained in EVs may have the capacity to modulate landmark signalling pathways involved in metabolism and cellular processes. To investigate this, we evaluated the impact of EVs released by MDV-tumour cells on the proliferation of chicken immune cells. Mononuclear cells were isolated from the bursa of Fabricius and spleens of healthy chickens. Primary B-lymphocytes were activated from bursal monocytes using recombinant soluble chCD40L, and T-cells were enriched from splenocytes by activation with an anti-TCR2 monoclonal antibody. EVs purified from 3867K supernatant were then added at different concentrations to B- and T-cells (activated or not). Cell proliferation was assessed by BrdU assay 24 h after EV exposure. We found that neither 10K-EVs nor 100K-EVs affected the proliferation of non-activated primary B-cells (Fig. 5a). In the absence of EVs, activation of B-cells with chCD40L resulted in a threefold increase in cell proliferation compared to non-activated B-cells. However, when activated B-cells were cultured in the presence of EVs, a marked reduction in proliferation was observed. This inhibitory effect occurred with both types of EVs and was dose-dependent in the case of 100K-EVs. In contrast, both 10K- and 100K-EVs triggered an increase (up to threefold) of TCR-2-activated splenic T-cell proliferation, without affecting non-activated cells (Fig. 5b). To determine which T-cell population was targeted by EVs, we analysed the rate of T-CD4^+^ and T-CD8^+^ cells in TCR2-activated splenocytes cultured in the absence or presence of 100K-EVs (Fig. 5c). It is worth noting that no differences in cell granularity were detected based on FSC/SSC analysis, regardless of EV exposure (Fig. S2). The percentage of T-CD8^+^ cells showed a slight decrease in the presence of 100K-EVs (from 20.9 to 18.4%). In contrast, the proportion of T-CD4^+^ cells within the splenocytes population increased 5.8-fold upon exposure to 100K-EVs. These results indicate that 100K-EVs released by MDV-transformed lymphocytes can promote the expansion of the T-CD4^+^ splenocytes population when activated.

## Discussion

In the present study, we provide a comprehensive analysis of the protein cargo contained within EVs derived from MDV-transformed T-cells and demonstrate that these vesicles exert immunomodulatory effects by influencing lymphocyte proliferation.

We first showed that cultures of MDV-transformed T-cells produced EVs, which we could isolate into two fractions based on their density: 10K-EVs and 100K-EVs. These vesicles exhibited markers commonly found in mammalian EVs [46]. TEM analysis revealed that the 10K fraction predominantly contained large vesicles, while the 100K fraction was enriched in smaller, round-shaped vesicles, consistent with the morphology recently described for exosomes derived from the MDV-transformed T-cell line MSB1 [36,47]. The distinct nature of the 10K and 100K vesicles was further confirmed by lipidomic analysis, which showed significant differences in lipid composition between the two EV populations, probably due to their cellular origins and biogenesis pathways [48]. It should be noted that an in-depth lipid characterization was not performed herein as it fell outside the scope of this study. Based on these phenotypic analyses, the 10K and 100K fractions appear to be enriched in vesicles exhibiting characteristics of MVs and EXOs, respectively.

Proteomic analysis revealed some differences in protein composition between 10K-EVs and 100K-EVs; however, *in silico* functional annotation did not highlight major differences in signalling pathways or biological processes potentially regulated by these proteins. It should be highlighted that due to the limited annotation of the chicken genome, we also extended our search to multiple taxa. For proteins identified outside *Gallus gallus*, we were nevertheless able to demonstrate that, in most cases, an avian homologue was indeed identified (see Tables S1, S2 and S3).

A key observation from the proteomic dataset obtained from EVs derived from the 3867 K T-cell line is the absence of MDV-encoded proteins. This finding contrasts with results from a previous study, which identified viral proteins in exosomes isolated from the serum of Marek’s disease-vaccinated and unvaccinated lymphoma-bearing chickens [35]. In that report, viral proteins were detected in exosomes at very low abundance, limited to the immediate early protein ICP4 and the two tegument proteins UL36 and UL47 [35]. These three viral proteins are predominantly expressed during the lytic phase of MDV infection and are not typically associated with latency (except UL36, since UL36 mRNA could be detected in two MDV-transformed T-cell lines [49]). In the present study, we isolated EVs from a T-cell line mainly composed of latently infected cells. The 3867 K cells exhibit indeed minimal spontaneous reactivation (~0.1%) and predominantly express the latent viral oncoprotein Meq and non-coding RNAs (vTR, miRNAs, LATs and circRNAs) [50,54]. This could explain why none of these lytic proteins were detected in EVs released by 3867 K cells. Considering the situation in EVs shed from EBV-latently infected cells that carry the latent oncoproteins LMP-1 and LMP-2A, one might have expected to detect the Meq oncoprotein in 3867K-EVs [55,56]. However, this was not the case, probably due to Meq’s relatively low expression in MDV-transformed cell lines.

We have made the striking observation that 100K-EVs derived from MDV-transformed cells reduced the proliferation of bursal B-cells. A recent study investigating the impact of TEX on B-cells also reported that TEX exert immunosuppressive effects by inhibiting B-cell proliferation [57]. Of note, the proliferation of splenocytes activated or not by chCD40L was not affected by 3867K-EVs (data not shown). One explanation could be the high proliferative capacity as well as the early stage of maturation of bursal cells, which may confer an increased sensitivity and reactivity to EVs [58]. Taking into account the critical role of the bursa of Fabricius in primary B lymphopoiesis and B-cell differentiation, we cannot exclude the possibility that the inhibition of bursal B-cell proliferation by EVs released by MDV tumour cells contributes to the immunosuppression observed at later stages of MDV infection [59].

On the other hand, we observed that EVs released by MDV-transformed lymphocytes significantly enhance the proliferation of T-cells pre-activated with anti-TCR2. More precisely, 3867K-EVs preferentially promote the proliferation of CD4^+^ T-cells, while they tend to slightly reduce the proliferation of CD8^+^ T lymphocytes. This finding is particularly interesting as CD4^+^ cells are the major target cells for MDV latency and lymphomagenesis. It also echoes a previous study showing that EVs released by human tumour cells can induce the expansion of CD4^+^ T-cells, notably Tregs, while simultaneously triggering apoptosis in activated anti-tumour CD8^+^ effector cells [60]. The effect of 3867K-EVs on T-cell proliferation may be mediated either through receptor/ligand signalling or via mechanisms requiring the internalization of EVs. It is worth noting that our proteomic analysis identified several proteins that could contribute to the observed modulation of cell proliferation. One can imagine that MHC class I and II molecules present in the 3867K-EVs could participate in the modulation of T-cell proliferation [61]. Previous studies have demonstrated that the ability of TEX to trigger apoptosis in CD8^+^ T-cells can also be partly attributed to the presence of MHC class I molecules [60,62]. Similarly, it is conceivable that miRNAs could also play a role by modulating the expression of genes involved in cell cycle progression and apoptosis [63,65]. A recent study identified viral miRNAs in exosomes released from the MDV-transformed cell line [36]. Moreover, we cannot exclude that the pro-proliferative effect of 3867K-EVs on CD4^+^ T-cells can be mediated by non-coding RNAs.

Next to their impact on cell proliferation, proteins contained in 3867K-EVs could also impact signalling pathways involved in tumourigenesis processes such as glycolysis, integrin, PI3-Akt, hypoxia, Ras or MAPK pathways. In addition, the GO annotation revealed proteins associated with the positive regulation of telomere maintenance via telomerase. Six identified proteins (TCP1, DKC1 and CCT2/5/7/8) play a major role in the folding and transport of the telomerase complex, in particular toward the Cajal bodies and telomeres [66]. The identification of proteins linked to telomere length regulation is of particular interest since telomerase activity is a key process in MDV-induced tumourigenesis [67]. We can also not exclude the possibility that, in addition to these proteins, the vesicles may contain viral miRNAs and vTR, which could also facilitate cellular transformation [67,69]. The proteomic analysis performed herein represents a thorough characterization of the EVs, intended to guide future studies aiming to dissect the specific contributions of individual cargo types. Further functional investigations, such as selective depletion or enrichment of EV components, will be necessary to establish causal relationships between EV content and cellular uptake outcomes.

Based on our data, it appears that EVs released by latently MDV-infected cells may pave the way to MDV-induced transformation. These EVs could enhance the proliferative capacity of MDV-susceptible CD4^+^ T-cells while simultaneously suppressing the expansion of immune cell populations with anti-tumoural functions, including CD8^+^ cytotoxic T lymphocytes and B-cells. This combined effect could thus foster a microenvironment conducive to viral oncogenesis.

## Supplementary material

10.1099/jgv.0.002191 Uncited Supplementary Material 1.

10.1099/jgv.0.002191 Uncited Supplementary Material 2.

## Abbreviations

CESCs: chicken embryonic skin cells
chAEc: primary aortic endothelial cells
chCD40L: chicken CD40 ligand
CV: coefficient of variation
EBV: Epstein–Barr virus
EGFR: Epithelial Growth Factor
emPAI: exponentially modified protein abundance index
EVs: extracellular vesicles
EXOs: exosomes
GO: gene ontology
IS: immunosuppression
KEGG: Kyoto encyclopedia of genes and genomes
KSHV: Kaposi’s sarcoma-associated herpesvirus
MDV: Marek’s disease virus
miRNAs: micro-RNA
MVB: multivesicular bodies
MVs: microvesicles
nanoLC-MS/MS: nanoflow liquid chromatography-MS/MS
NCBI: National Center for Biotechnology Information
RT: room temperature
SPF: specific-pathogen-free
TEM: transmission electron microscopy
TEX: tumour-derived exosomes
vTR: viral telomerase RNA
