# Supplementary material for: Extracellular vesicles released from Marek’s disease virus-transformed T-cells impact immune cell proliferation
Source: J Gen Virol. 2025 Dec 4;106(12):002191. doi: 10.1099/jgv.0.002191 (PMC12677464; doi:10.1099/jgv.0.002191)
Supplement: Uncited Supplementary Material 1. [file jgv-106-02191-s001.pdf]

Figure S1

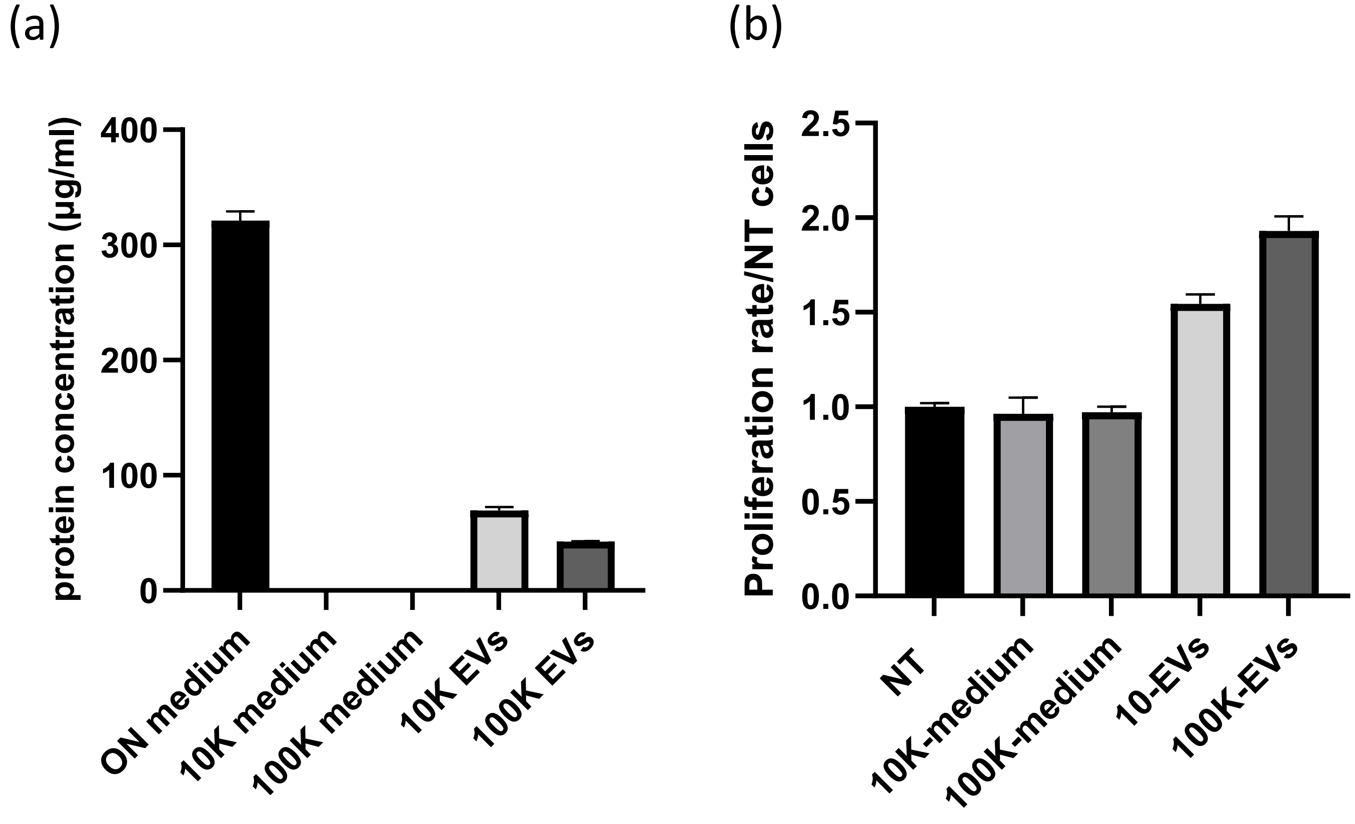

Figure S1. Control assays to test the presence of contaminants in medium

To generate EV-depleted medium, RPMI 1640 supplemented with 10% FBS was subjected to overnight ultracentrifugation at 110,000 × g using a 45Ti rotor at 4°C. Given that the serum used in this preparation likely contains extracellular vesicles (EVs), the resulting pellet from this ultracentrifugation was resuspended in 400 µl of PBS1X and designated as the positive control (ON medium). The supernatant, presumed to be depleted of EVs, was then processed through the same differential ultracentrifugation protocol employed for EV isolation from culture supernatants. This procedure yielded two distinct fractions: (i) the 10K pellet and (ii) the 100K pellet, both of which were resuspended in 400 µl of PBS1X. In parallel, 10K and 100K EVs were isolated from the supernatant of 3867K cells, and the corresponding pellets were similarly resuspended in 400 µl of PBS1X. (A) Protein quantification using Micro BCA assays (Thermo Scientific) showed no detectable protein in the 10K- and 100K-medium (same optical density as the PBS1X blank), while the initial pellet (ON medium) and 10K-, 100K-EVs contained 321.2 µg/ml, 69.5 µg/ml and 42.3 µg/ml, respectively. (B) We further evaluated whether the conditioned media might contain residual serum-derived contaminants that could potentially interfere with downstream biological assays. Stimulated splenocytes were cultured for 24h with 8µg of 10K- and 100K- EVs or the equivalent volume of 10K- and 100K-media fractions. Cell proliferation was assessed using BrdU incorporation. The fractions obtained from the conditioned media alone did not elicit splenocyte proliferation, in contrast to the 10K-EVs and 100K-EVs obtained from the 3867K supernatant. These results demonstrate that the “exo-free” medium used for EV isolation is effectively devoid of EVs and other contaminating factors that might have induced artefactual proliferation.

Figure S2

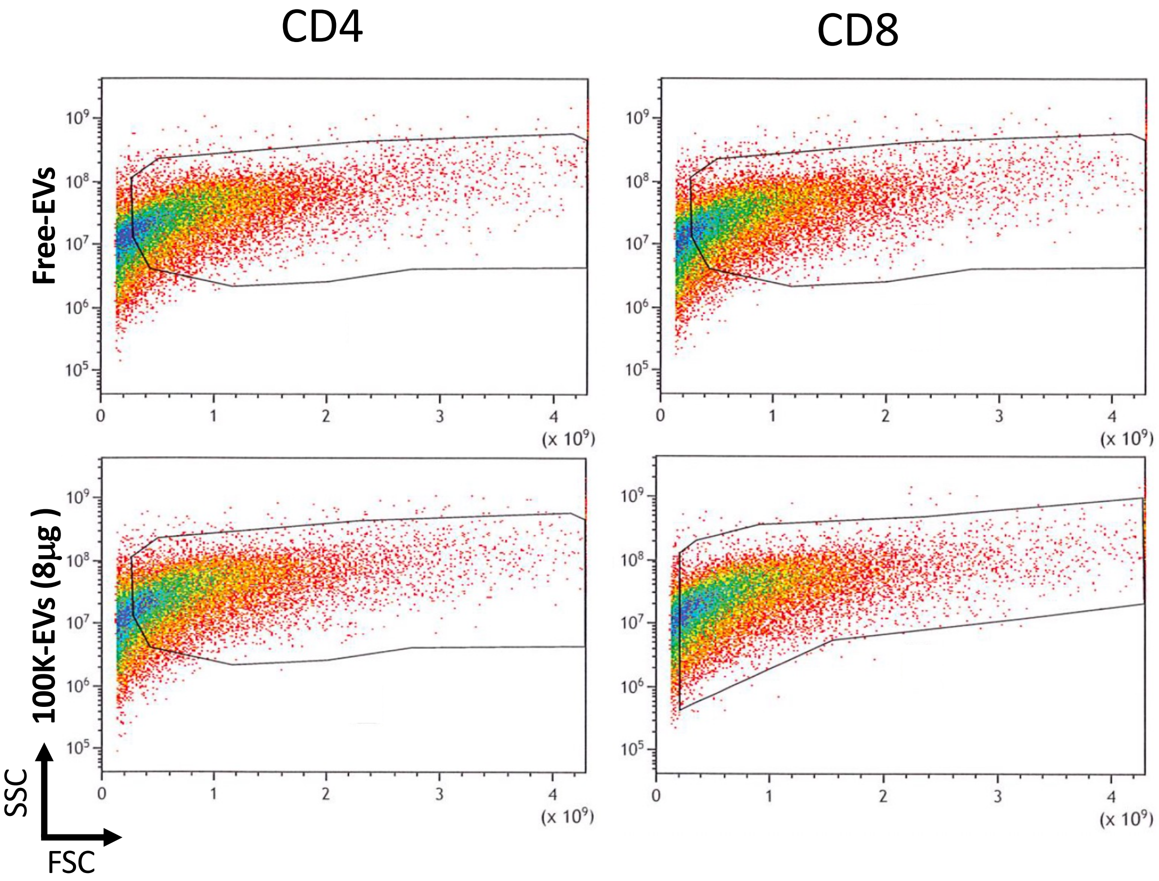

Figure S2. Impact of 3867K-EVs on T-cells granularity

Eight micrograms of 100K-EVs, isolated from 3867K supernatant, were either added to TCR2-activated splenocytes or omitted as a negative control. After 24 hours of incubation, the expression of CD4 and CD8 T-cell markers was assessed via immunostaining followed by flow cytometry analysis. Cell granularity was evaluated using side scatter (SSC) and forward scatter (FSC) parameters. Representative dot plot diagrams from two independent experiments are shown. No differences in cell granularity were observed between conditions, regardless of EV presence in the culture.
